# A systematic review of psychological interventions in total hip and knee arthroplasty

**DOI:** 10.1186/s12891-018-2121-8

**Published:** 2018-06-21

**Authors:** Samantha Bay, Lukas Kuster, Neil McLean, Michelle Byrnes, Markus Stefan Kuster

**Affiliations:** 1 M304, School of Psychological Science, The University of Western Australia, 35 Stirling Highway, Perth, Western Australia 6009 Australia; 2 Sir Charles Gairdner Hospital, Hospital Ave, Perth, Western Australia 6009 Australia; 3 Perron Institute for Neurological and Translational Science, The University of Western Australia, RR Block, QEII Medical Centre, 8 Verdun Street, Perth, Western Australia 6009 Australia; 4 Medical School, The University of Western Australia, 35 Stirling Hwy, Perth, Western Australia 6009 Australia

**Keywords:** Arthroplasty, Knee, Hip, Psychology, Intervention

## Abstract

**Background:**

The current practice in elective orthopaedics does not routinely include psychological interventions despite evidence that psychological factors such as personality, anxiety, depression and negative thinking styles can influence outcomes and recovery from surgery. The objective of this paper was to review the effectiveness of psychological interventions used in conjunction with total hip (THA) and knee arthroplasty (TKA), in improving patient reported joint outcomes.

**Methods:**

An extensive literature search was conducted according to Preferred Reporting Items for Systematic reviews and Meta-Analyses guidelines. Search terms included psychology, interventions, and orthopaedics. Articles were included if they were randomised controlled trials (RCTs) of psychological interventions involving active patient participation measured with patient reported joint outcomes in patients undergoing hip or knee arthroplasty.

**Results:**

A total of 19,489 titles were screened. Seven studies met the inclusion criteria and were included. Five of seven studies did not show improvements in patient reported outcomes after surgery. Specifically, psycho-education alone was not effective at improving patient reported joint outcomes in two out of two studies.

**Conclusion:**

The current literature does not support routine psychological interventions for TKA and THA. However, it should be noted that the literature for psychological interventions in conjunction with TKA and THA is still in its infancy. This gap in the literature is surprising, considering the importance of the role of psychological factors in recovery. Further RCTs with long term follow ups, multidisciplinary involvement, and more comprehensive and focused interventions that go beyond educating patients are needed. Future studies should account for the demand effect, include measures of psychological variables to determine whether psychological interventions are more beneficial for some patients compared to others, and compare the different modes of delivery and timing of interventions to determine the optimal nature and duration of psychological interventions for TKA and THA.

## Background

The current practice in elective orthopaedics does not involve routine psychological interventions, despite evidence that psychological factors influence outcome and recovery from surgery [1–3]. One in eight patients experience moderate to severe levels of pain one year after total knee arthroplasty (TKA) despite having normal clinical and radiographic findings [4]. Many studies have suggested that psychological factors such as personality, anxiety, depression and negative thinking styles influence outcomes and recovery from surgery [1–3, 5, 6]. Giesinger et al. documented that psychological and demographic factors accounted for more variance in patient reported outcomes after hip and knee arthroplasty, than surgical factors [1]. Therefore, it would seem logical that inclusion of psychological interventions to facilitate recovery from arthroplasty may enhance patient satisfaction and outcomes, as psychological factors can influence perception of pain, participation in rehabilitation and other outcomes after surgery [7].

Previous meta-analyses and systematic reviews [8–10] of psychological interventions found some to be effective in improving physical and psychological outcomes after surgeries. For example, in the most recent review, Nelson et al. investigated 20 studies with patients undergoing abdominal, cardiac, and orthopaedic surgery, and documented that there was some evidence for relaxation therapy in improving psychological well-being, such as reducing tension, anger, anxiety and pain, and evidence that guided imagery reduced post-surgical pain levels, and reducing analgesic intake [9].

Most reviews include a wide range of surgical procedures, which makes it difficult to draw conclusions and frame recommendations specific to TKA and total hip arthroplasty (THA). Arthroplasty is an elective surgery and is often undergone by healthy individuals with relatively low rates of comorbidities [11], and is thus very different from other surgeries, for example, coronary artery bypass grafting where patients require surgery in order to prolong life.

This systematic review aimed to bring more clarity with respect to the effectiveness of psychological interventions in improving joint outcomes following hip and knee arthroplasty. The following questions were addressed: Are psychological interventions beneficial in improving recovery and joint outcomes after TKA and THA? If so, are all types of psychological interventions equally effective?

## Descriptions of psychological interventions in reviewed articles:

Cognitive behaviour therapy, psycho-education, motivational interviewing, relaxation therapy and guided imagery are some examples of well-established evidence-based psychological therapies in the literature.

### Cognitive behaviour therapy

Cognitive behaviour therapy seeks to reduce symptoms by modifying maladaptive thought patterns and behaviours [12, 13]. It is based on the works of Beck [14] and Ellis [15]. Ellis proposed the ABC model of irrational beliefs, which cognitive behaviour therapy is based upon [16]. In the ABC model, an activating event (A), such as pain after surgery, in combination with a negative/irrational Belief (B), thinking that surgery is only successful if there is no pain at all, leads to a maladaptive behavioural or cognitive consequence (C), thinking that the pain will last forever and that the procedure had failed [16]. Cognitive behaviour therapy aims to help the patient understand and alter beliefs and thought processes, in order to positively influence consequences [15, 17, 18]. The therapist guides the patient to actively recognise maladaptive beliefs and thoughts, and to effect changes in emotional and behavioural consequences [18].

### Psycho-education

Psycho-education is the systematic education of patients about their condition, along with discussion of coping strategies that might be used to manage and cope with current and future problems [19–21]. Psycho-education is often part of cognitive behaviour therapy [22, 23], but unlike cognitive behaviour therapy, Psycho-education does not aim to change emotional and behavioural consequences by exploring and changing a patient’s belief, but rather gives the patient information about their condition and offers suggestions of behaviour changes that they can implement when faced with problems.

### Motivational interviewing

Motivational interviewing is a counselling style that is targeted at eliciting behaviour changes [24]. Unlike persuasion, which generally increases resistance from the patient, motivational interviewing aims to explore and resolve the patient’s ambiguity to change [24]. The counsellor uses empathy and acknowledges resistance, to guide the patient to create a discrepancy between reasons for and against change [25]. It is paramount that the patient reaches the conclusion on their own accord, with guidance from the counsellor [25].

### Relaxation therapy

Relaxation therapy encompasses a range of techniques designed to reduce muscle tension and autonomic arousal [26]. This is achieved by using skills that focus on the internal state of the individual such as controlled breathing, focused muscle relaxation and postural awareness and management. [26]. Techniques typically focus on the redirection of attention of the patient from their thoughts and emotions [27].

### Guided imagery

Guided imagery is a type of relaxation therapy in which patients deliberately form mental representations of positive images to promote relaxation and body awareness [28, 29]. While many relaxation therapies focus on physical components such as breathing and muscle tension, guided imagery most commonly focuses on sensory information such as sound, smell, touch, vision and taste [29, 30].

## Methods

Preferred Reporting Items for Systematic reviews and Meta-Analyses (PRISMA) guidelines were applied. There was no published protocol for this review.

### Search strategy

The search included empirical articles published in peer-reviewed journals, conference abstracts, and unpublished articles between January 1980 and mid-May 2017. An extensive literature search was conducted by searching electronic databases (Keyword and MeSH explode) for published articles and conference abstracts (MEDLINE, PsycInfo, EBSCO, PubMed, CINAHL, Web of Science, Scopus), grey literature (PsycExtra, Cochrane Library), and dissertations and theses (ProQuest Dissertations and Theses). Hand-searching was also conducted by reviewing the references cited in previous systematic reviews, and articles included in this study.

**Fig. 1:**
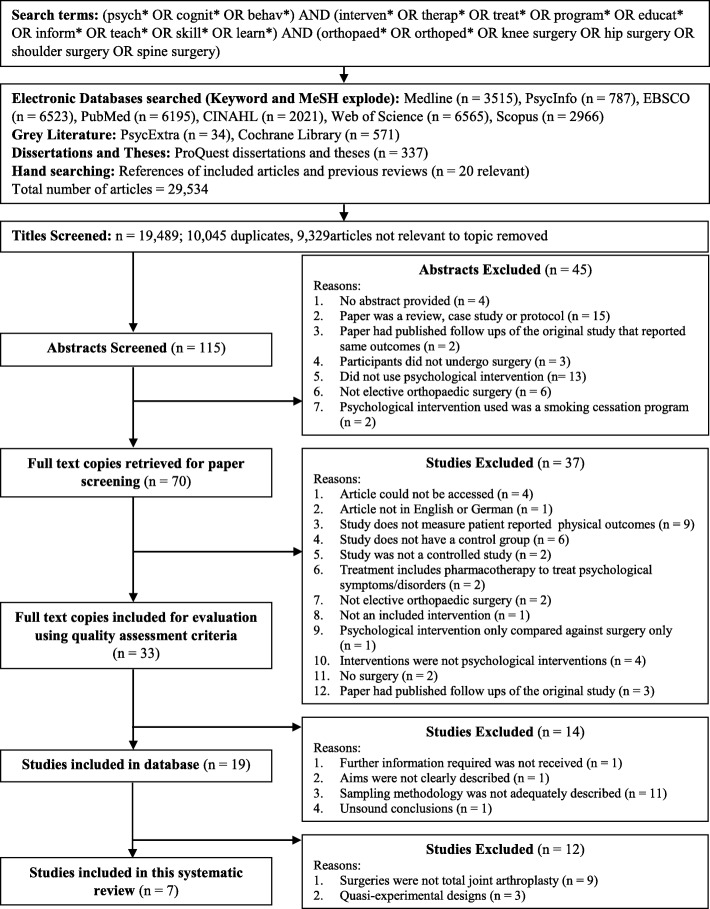
Flow chart of search, retrieval and inclusion process

Unpublished dissertations, theses and grey literature were included in the search in order to avoid publication bias. Authors of conference abstracts, who reported collecting data on patient reported outcomes in elective orthopaedic surgery after psychological interventions were contacted by email for further information about their studies. The date of the last search was 17 May 2017. The search methodology is detailed in Fig. 1.

The following search terms were used: (psychological OR cognitive OR behavioural) AND (intervention OR therapy OR treatment OR program OR education OR information OR teaching OR skill OR learning) AND (orthopaedic OR orthopedic OR knee surgery OR hip surgery OR shoulder surgery OR spine surgery). Boolean truncations were used to allow for a more expansive search (see in Appendix for an example).

The terms chosen covered were broad in focus to allow inclusion of all studies that investigated the role of psychological intervention in elective orthopaedic surgery. However, in order to understand the more specific effects of psychological interventions on patients undergoing hip and knee arthroplasty, articles included in this review were further restricted.

### Selection criteria

Studies included in this review satisfied the PICOS criteria:

**P**articipants: elective orthopaedic patients undergoing knee or hip arthroplasty;

**I**nterventions: psychological interventions involving active patient participation;

**C**omparisons: control groups including placebos, sham treatments, treatment as usual, education only or physical exercise only;

**O**utcome: Patient reported joint outcomes including pain, and/or functionality, and/or disability;

**S**tudy designs: Randomised controlled trials (RCTs).

Studies were excluded if participants did not undergo surgery, and/or if psychological intervention was compared to surgery. Studies were also excluded if they did not include a control group, and if a patient reported joint outcome was not measured. Articles that were in languages other than English and German were excluded. Only randomised control trials were reviewed.

### Quality assessment

Only articles that fulfilled the requirements of the quality assessment were included in the review. In order to be considered good quality, an article must: 1) clearly describe the aims of the study; 2) adequately describe the methods undertaken in the study such that it may be replicated, which required clear descriptions of the recruitment of participants, timeline of interventions and data collection; and 3) have logical and sound conclusions drawn from results of the study.

Two authors (SB and LK) conducted the systematic searches and reviewed articles independently, according to the selection criteria. When there were disagreements regarding whether an article should be included, the issue was discussed with a third author (MSK), and a decision was made when all authors came to an agreement.

### Data extraction

Data on participants, type of surgery, type of intervention, intervention timeline, sample size, follow ups, drop outs, outcome measures, professional backgrounds of therapists, power calculations, and major findings regarding patient reported joint outcomes were documented on pre-designed spreadsheets. Interventions were classified as effective if at least one outcome related to the arthroplasty was significantly improved for the treatment group compared to control group, after surgery. Data was extracted by the first author and checked by the second author.

### Risk of Bias assessment

All included papers were reviewed for risk of bias, by assessing seven criteria adapted from the Cochrane Risk of Bias Tool [31]: 1) adequate randomization (selection bias); 2) dropout rate was not a threat to power (attrition bias); 3) demand effect - assessor of outcomes should not be the therapist/clinician (detection bias); 4) complete reporting of outcome data in results section; 5) significant and non-significant findings reported appropriately in the discussion section (reporting bias); 6) monitored intervention integrity; and 7) appropriate use of statistical analyses. Advice from a statistician about the appropriateness of statistical analyses was obtained.

There were two stages in defining risk of bias. Firstly, the articles were assessed according to the seven criteria, and rated with a risk score of “high” or “low” for each criterion. If there was inadequate information in articles to determine whether a criterion was satisfied, then a risk score of “high” was given. Secondly, a pre-defined classification of overall risk of bias assessment was agreed upon by the authors. Each article was rated to have either low, moderate or high overall levels of risk of bias, according to the following classification: low risk (A) where the article satisfies all 7 criteria in stage one; moderate risk (B) where 1–3 criterion were not satisfied in stage one; and high risk (C) where more than 3 criterion were not satisfied in stage one.

## Results

### Study selection

A total of 19,489 titles of studies were screened. Seven studies met criteria, and were included in this review (Fig. 1).

### Study characteristics

Characteristics of the included studies are presented in Table 1.

**Table 1:** Summary of reviewed studies

Author (Year)	Participants	Type of surgery	Control group	Psychological intervention (Type)	Frequency of sessions + (Mode of delivery)	Pre or Post surgery	Data collection points	Outcome measures (Note: Patient reported joint outcomes in bold)	Main findings about patient reported joint outcomes
Berge, Dolin, Williams & Harman (2004) [32]	Total *N* = 44 Mean age = 71.6 (PMP), 71.0 (controls) Age range = NS	THA	*N* = 21 TAU	Pain Management Training: education, cognitive behaviour therapy, relaxation **(CBT + RT)**	Total = 21.5 h; Occurred over 6 weeks; 1 to 2 mornings a week **(In-person)**	Pre; at least 6 months before surgery	Total = 3; Pre intervention, 3 and 12 months after PMP	**Pain (numerical rating scales)**, Analgesic drug use, **function (Arthritis Impact Measurement Scale),** metres walked in 4 min	Treatment group had sig. Better function than controls at 12 month follow up. No sig. Difference in pain was found between groups at 12 month follow up.
Doering et al. (2000) [33]	Total *N* = 100 Mean age = 58.7 (treatment), 60.4 (control) Age range = NS	THA	*N* = 54 TAU	Educational video + discussion **(PE)**	Total = 1 × 12 min video watched with psychologist/psychiatrist **(Video recording)**	Pre: afternoon of the pre-operative day	Total = 8; 5 consecutive days starting on pre-operation day, 3 months post-surgery	Anxiety (STAI), Depression (Von Zerssen Depression Scale), **Pain (VAS),** Blood pressure, Heart rate, Stress hormones (Urine samples), intake of analgesics and sedatives	No sig. Difference in pain was found between groups at all time points.
Forward et al. (2015) [38]	Total N = 224 TKA = 154 THA = 70 Mean age = NS Age range = NS	THA + TKA	*N* = 75 TAU TKA = NS THA = NS	Guided Imagery: Guided Meditation for Procedures or Surgery **(GI)**	Total = 4 × 18-20 min sessions: pre-surgery day, post-surgery day 0, 1 and 2 **(Audio recording)**	Pre and Post: began after admission	Total = 8; pre and post intervention on pre-surgery day, post-surgery day 0, 1 and 2	**Pain (Numeric Rating Scale)**, Anxiety (Numeric Visual Anxiety Scale, Hamilton Anxiety Scale)	No sig. Differences in pain between GI and controls during overall hospital stay.
Frost (2003) [34]	Total *N* = 24 Mean age = 66.2 (treatment), 65.9 (control) Age range = 57–75	THA	*N* = 11 Telephone contact only	Motivational Interviewing **(MI)**	Total = 3 sessions; 1 × 1 h in person, 2 x bi-monthly 15-30 min telephone session **(In-person and over the telephone)**	Post: began 3 months post-surgery	Total = 2; Pre intervention, 5 months post-surgery	Exercise (diary), Physical activity (Modifiable Activity Questionnaire), Mobility (Gait Speed), Muscle force/balance (Timed Chair Rise), **Pain/Stiffness/physical function (WOMAC), physical health and mental health (SF-36),** Self-efficacy (Self Efficacy for Exercise Questionnaire), Depression (CESD-10)	No sig. Differences found between groups for all measures at 5 months post-surgery.
Grossman (2016) [37]	Total *N* = 60 TKA = 44 THA = 16 Mean age = 66.1 Age range = NS	THA + TKA	*N* = 15 Education only TKA = 10 THA = 5	Guided Imagery **(GI)**	Total = not standardised: 6 min recording pre-surgery twice a day for 1–2 weeks (At least once on day of surgery), encouraged to listen to recording post-surgery but no specific instruction. **(Audio recording)**	Pre: before admission and before surgery	Total = 3; pre intervention, post intervention (during hospital admission), post-surgery (before discharge)	Anxiety (STAI-S), Stress (Perceived Stress Scale), Coping Strategies (CSQ), **Pain (VAS)**, compliance (Training survey), medication use, length of hospital admission	No sig. Differences between groups for pain overall.
Jacobson et al. (2016) [36]	Total *N* = 82 Mean age = 65.0 Age range = 41–81	TKA	*N* = 40 Placebo audio recordings	Guided Imagery **(GI)**	Total = 35 self-directed sessions; Every day for 2 weeks before, and 3 weeks after surgery; 19-21 min each session for treatment, 17-21 min each session for control **(Audio recording)**	Pre and post: began 2 weeks pre- surgery	Total = 4; 2-3 weeks before surgery (pre-intervention), day of surgery, 3 weeks after surgery (post-intervention), 6 months after surgery	Gait velocity (Timed 10-Meter Walk), **functional status (SF-36), Pain/stiffness/function (WOMAC),** imaging ability (Imaging Ability Questionnaire), optimism/pessimism (TKR Outcome Expectancy), **daily pain (VAS)**, self-efficacy (Self-Efficacy for Rehabilitation Scale), pain catastrophizing (PCS), fear of movement (Tampa Kinesophobia Scale), CD use questionnaire, Physiological variables (Lymphocytes, macrophages and cytokines)	Treatment group had sig. Reduced pain levels compared to baseline 3 weeks after surgery than control group. No sig. Differences between groups in improvements of knee function at 6 months post-surgery.
McGregor et al. (2004) [35]	Total *N* = 39 Mean age = 71.9 Age range = 51–92	THA	*N* = 20 TAU	Education + discussion **(PE)**	Total = 1 x advise class **(In-person)**	Pre surgery: 2-4 weeks before surgery	Total = 4; Pre-intervention, at admission, at discharge, 3 months post-surgery	**Function (WOMAC, Harris Hip Score, Berthel Activities of Daily Living Index), Pain (VAS),** Mood states (Positive Affect Negative Affect Scale), helplessness (subscale of Rheumatology Attitudes Index), **Fatigue (VAS),** expectations of pain/function/satisfaction (VAS), Life satisfaction (Cantril Life Satisfaction Ladder)	No sig. Difference was found between groups for pain and function 3 months post-surgery. No results were reported for differences between groups in fatigue.

*Note. “NS” indicates information not specified by papers. The word “significant” was abbreviated with “sig.”. CBT = Cognitive Behaviour Therapy, MI = Motivational Interviewing, GI = Guided Imagery, PE = Psycho-education, RT = Relaxation Therapy, TAU = Treatment as usual, PMP = Pain management program, STAI = State-Trait Anxiety Inventory, VAS = visual analogue scale, WOMAC = Western Ontario and McMaster Universities Osteoarthritis Index, PCS = Pain Catastrophizing Scale, CESD-10 = The Centre for Epidemiologic Studies Depression scale 10*

#### Participants

The 7 reviewed studies consisted of 7 randomized controlled trials [32–38]. A total of 573 participants were involved, where 280 participants underwent a total knee arthroplasty (TKA), while 293 underwent a total hip arthroplasty (THA).

#### Analysis of studies

The data extracted from studies were categorised according to effectiveness in improving patient reported outcomes, and are presented in Table 2. Table 2 also summarises the joint outcomes that were found to be improved by interventions, and joint outcomes that were not found to be improved by interventions.

**Table 2:** Effectiveness of interventions in improving patient reported outcomes after surgery

*Not effective*					Effective					
*Study*	*Psychological intervention (Surgery)*	*Mode of delivery*	*Timing of Intervention*	*Joint outcomes measured*	Study	Psychological intervention (Surgery)	Mode of delivery	Timing of intervention	Joint outcome improved	Joint outcomes measured but not sig.
*Doering* et al. *(2000)* [33]	*Psycho-education* *(THA)*	*Video*	*Pre-surgery*	*Pain*	Berge et al. (2004) [32]	CBT + Relaxation (THA)	In-person	Pre-surgery	Function	*Pain*
*Grossman (2016)* [37]	*Guided Imagery* *(THA + TKA)*	*Audio*	*Pre-surgery*	*Pain*	Jacobson et al. (2016) [36]	Guided Imagery (TKA)	Audio	Pre- and Post-surgery	Pain	*Function, Stiffness*
*Frost (2003)* [34]	*Motivational Interviewing* *(THA)*	*In-Person + Telephone*	*Post-surgery*	*Pain, Stiffness, Function, Physical Health*						
*Forward* et al. *(2015)* [38]	*Guided Imagery* *(THA + TKA)*	*Audio*	*Pre- and post-surgery*	*Pain*						
*McGregor* et al. *(2004)* [35]	*Psycho-education* *(THA)*	*In-person*	*Pre-surgery*	*Function, Pain*						

*Note. The word “significant” was abbreviated with “sig.”*

#### Types of psychological interventions

Two studies used psycho-education [33, 35], one used motivational interviewing [34], and three used guided imagery [36–38]. One study used a combination of cognitive behaviour therapy and relaxation therapy [32].

#### Effectiveness of interventions

Patient reported outcomes included pain, physical health status, physical function, stiffness and fatigue. The most common parameter was pain, either measured on a numerical rating scale, visual analogue rating scale (VAS), or as part of the Western Ontario and McMaster Universities Arthritis Index (WOMAC).

Overall, two out of seven studies (total *n* = 126, 65 treated) found psychological interventions to be effective in improving at least one patient reported joint outcome [32, 36]. Of these two studies, one study (total *n* = 44, 23 treated) used a combination of cognitive behaviour therapy and relaxation therapy and was found to significantly improve hip function at the 12 month follow-up [32], and one study (total *n* = 82, 42 treated) used guided imagery and was found to significantly decrease knee pain 3 weeks after surgery [36]. Psycho-education, guided imagery, and motivational interviewing were amongst the types of interventions that were found to be ineffective in improving patient outcomes after TKA and THA.

The number of sessions of interventions varied widely, ranging from 1 to 35 sessions. Interventions that were effective in improving patient reported joint outcomes ranged from 6 to 35 sessions [32, 36]. All interventions with less than six sessions were not effective in improving patient reported outcomes [33–35, 38], and one intervention with more than 14 sessions was found to be ineffective [37]. However one intervention with 6 to 12 sessions [32] and another intervention with 35 self-directed sessions [36] were found to improve patient reported outcomes.

#### Mode of delivery

Mode of delivery varied across the studies with interventions delivered face to face, by video, by audio and by a mix of telephone and face to face contact. Effective interventions were delivered face to face [32], and by audio recording [36]. However, not all interventions delivered face to face and by audio recording were effective. Of two studies that delivered psychological interventions in-person (i.e. face to face) [32, 35], one [32] was effective (total *n* = 44, 23 treated). One study delivered interventions partially over the telephone and partially in-person [34], and was found to be ineffective. One study used video recordings [33], and was found to be ineffective. Three studies used audio recordings to deliver psychological interventions [36–38], and one [36] was effective (total *n* = 82, 42 treated).

#### Timing of interventions

Interventions were delivered either pre-surgery, post-surgery, or both pre and post-surgery, and varied considerably in terms of the number of sessions conducted. Four studies delivered psychological intervention sessions pre-surgery [32, 33, 35, 37], while one study delivered sessions post-surgery [34]. The number of sessions in pre-surgery interventions ranged from 1 to 28 sessions [32, 33, 35, 37], while the post-surgery intervention had 3 sessions [34]. Two studies [36, 38] delivered psychological intervention sessions both pre-surgery and post-surgery. The number of pre-surgery sessions ranged from 1 to 14 sessions, and post-surgery sessions ranged from 3 to 21 sessions [36, 38].

There was no clear trend as to the effectiveness of interventions according to the timing in which interventions were delivered. One intervention that was effective in improving at least one patient-reported outcome after surgery [36] was delivered both pre- and post-surgery (total *n* = 82, 42 treated), while the other intervention that was effective (total *n* = 44, 23 treated) was delivered pre-surgery [32]. The intervention delivered post-surgery only was not found to be effective [34], the other intervention delivered both pre and post-surgery was found to be ineffective [38], and the other three interventions delivered pre-surgery only were found to be ineffective [33, 35, 37].

#### Length of follow up

The timing of follow up measurements was variable and ranged between 1 day and 12 months post-surgery. One study conducted a follow up 12 months post-surgery [32], one study at 6 months post-surgery [36], one study at 5 months post-surgery [34], two studies at 3 months post-surgery [33, 35], and two studies conducted follow ups less than 1 week post-surgery [37, 38].

### Risk of bias across studies

Of 7 studies, 5 had moderate risk of bias [32–34, 36, 38] and 2 had high risk of bias [35, 37].

The most adhered to criteria were adequate randomization and complete reporting of findings in the discussion section. The least adhered to criterion was having an independent researcher (i.e. not therapist or clinician) to collect outcome measures, which increased detection bias. As expected, none of the studies blinded participants or clinicians providing the interventions, as it is not possible due to the nature of psychological interventions. A summary of the risk of bias assessment is displayed in Table 3.

**Table 3:** *Risk of bias assessment*

Authors	Risk of bias	Selection bias	Attrition bias	Detection bias	Complete outcome data (results)	Reporting bias	Compromised Intervention integrity?	Appropriate use of statistics?
Berge et al. (2004) [32]	B	Low risk	**High risk**	**High risk**	Yes	Low risk	**High risk**	Yes
Doering et al. (2000) [33]	B	Low risk	Low risk	**High risk**	**No**	Low risk	Low risk	Yes
Forward et al. (2015) [38]	B	Low risk	Low risk	**High risk**	**No**	**High risk**	Low risk	Yes
Frost (2003) [34]	B	Low risk	**High risk**	Low risk	Yes	Low risk	Low risk	Yes
Grossman (2016) [37]	C	Low risk	**High risk**	**High risk**	Yes	Low risk	**High risk**	**No**
Jacobson et al. (2016) [36]	B	Low risk	**High risk**	Low risk	Yes	Low risk	Low risk	**No**
McGregor et al. (2004) [35]	C	**High risk**	Low risk	**High risk**	**No**	Low risk	**High risk**	Yes

*Note. Overall risk of bias: low risk (A), moderate risk (B) and high risk (C). Highlighted cells indicate unsatisfied criterion. No = high risk*

## Discussion

Five out of seven RCTs did not show a benefit for psychological interventions in TKA and THA, questioning whether psychological interventions should be part of routine arthroplasty surgery. However, it should be noted that the literature for psychological interventions in conjunction with TKA and THA is still in its infancy, considering that 10 data-bases were searched, and only 7 RCTs met criteria to be reviewed. Many studies had small sample sizes and moderate levels of risk of bias despite being RCTs. The sub-optimal quality of articles exploring the effects of psychological interventions on patient reported joint outcomes is concerning, and the gap in the literature is surprising, considering the importance of the role of psychological factors in recovery.

Across the 7 studies, many different types of interventions were applied, and the timing and mode of delivery was variable, making comparisons difficult. Despite this, some interesting conclusions and directions for future research can be drawn.

Firstly, psycho-education was found to be ineffective in improving patient reported joint outcomes. Both RCTs applying psycho-education only found no significant differences between treatment and control groups [33, 35]. Imparting information alone to patients, while necessary, seems insufficient to change behaviour and therefore does not improve outcomes. This is in keeping with previous findings [39, 40]. Many studies have shown that psychological factors such as personality, anxiety, depression, and negative thinking styles influence the outcomes after surgeries [1–3, 5, 6]. Given that psycho-education alone is not effective in improving patient-reported joint related outcomes after surgery, these psychological factors need to be addressed using more comprehensive interventions that go beyond educating patients. In other words, changing patients’ thinking styles and providing strategies for managing physiological states are important in achieving improved patient reported outcomes after TKA and THA.

Secondly, interventions with less than six sessions were found to be ineffective, but beyond this, there was no clear relationship between the number of sessions and effectiveness of interventions. There was also no clear indication for the effectiveness of the different modes of delivery, or timing of interventions (i.e. pre/post-surgery). Further research is needed to define the optimal nature and duration of psychological intervention for TKA and THA.

There was promising evidence from two RCTs which integrated some form of relaxation therapy in their interventions, that psychological interventions improved outcomes post-surgery [32, 36]. Both studies showed a lasting effect beyond the allocated therapy period. One study that delivered cognitive behaviour therapy and relaxation therapy with a clinical psychologist found improvements in patient-reported functionality 12 months after surgery [32], and the other study found that guided imagery improved patient-reported pain 3 weeks after surgery [36].

### Limitations

This systematic review identified a growing body of literature that explored the use of psychological interventions in TKA and THA. The quality of studies was sub-optimal with various sources of bias identified. Most studies did not account for a demand effect, where therapists or clinicians that administered interventions also collected outcome data. The demand effect is related to response-biases, where patients respond to questions in order to maintain socially desirable appearances [41]. It is possible that patients report improvements in order not to disappoint their therapist, or to maintain an image of being a “good patient”. Future studies can account for the demand effect by having an independent researcher administer the questionnaires to patients and by including objective measures of outcomes, for example, measuring the range of motion to assess functionality of the knee or hip.

One of the main limitations of the current literature is the lack of long-term follow ups. Many studies focused on early postoperative outcomes, often only exploring outcomes during hospitalisation. It has been found that majority of patients experience low to mild levels of pain immediately after TKA [42], which mostly declines to half the intensity after 3 months [4]. Subjective perception of pain and functionality of the knee improves over a period of two years, and reaches a plateau after this period [43]. Thus, it would be important for studies to monitor patient outcomes over a longer period, as the rate of recovery varies over months [4, 42, 43].

Only one study in this review conducted a follow up at 12 months post-surgery [32] and one study conducted a follow up at 6 months post-surgery [36]. Thus, a conclusion as to whether psychological interventions have a lasting influence on patient outcomes cannot be drawn.

Most studies in the literature did not have specific intervention programs targeting different groups of patients in recovery from TKA and THA, rather, they were implemented as general concepts to every patient. Many patients will do well after TKA and THA, and psychological interventions may be more beneficial for patients with higher levels of catastrophic thinking styles, depression or anxiety as patients with these traits tend to have worse outcomes after surgery [1–3, 5, 6]. Future studies may wish to include measures of these psychological variables, to gauge whether psychological interventions are more beneficial for some patients compared to others. Additionally, most studies lacked a multi-disciplinary approach, where there was a lack of input from both mental health practitioners (e.g. psychologist or psychiatrist) and a surgical team member (e.g. surgeon). Future studies may wish to explore whether psychological interventions targeting recovery from TKA and THA specifically, with involvement of a multi-disciplinary team are effective.

## Conclusions

The current literature does not support the effectiveness of psychological interventions in improving patient reported joint outcomes after TKA and THA as most interventions explored by studies were found to be ineffective. Specifically, psycho-education alone was shown to be ineffective. It should be noted that the literature for psychological interventions in conjunction with TKA and THA is still in its infancy. This gap in the literature is surprising, considering the importance of the role of psychological factors in recovery. Further RCTs with long term follow ups (e.g. at least 1 year), with more comprehensive and focused interventions that go beyond educating patients are needed. Future studies should account for the demand effect by involving an independent researcher and including objective measures of joint outcomes, include measures of psychological variables to determine whether psychological interventions are more beneficial for some patients compared to others, involve a multidisciplinary team, and compare the different modes of delivery and timing of interventions to determine the optimal nature and duration of psychological interventions for TKA and THA.

## Appendix

**Table 4:** Example of search terms

Search terms in MEDLINE	
All fields Limit to yr. = “1980–2017”	(Psychiatry/ or psych*.mp. OR exp. Cognition/ or exp. Cognitive Processes/ or cognit*.mp. OR behav*.mp. or exp. Behavior/) AND
	(interven*.mp. OR exp. Psychotherapy/ OR therap*.mp. OR exp. Treatment/ or treat*.mp. OR program*.mp. OR educat*.mp. OR exp. Information/ or inform*.mp. OR exp. Teaching/ or teach*.mp. OR exp. Skill learning/ or skill*.mp. OR exp. Learning/ or learn*.mp.) AND
	(Orthopedics/ or Orthopedic Procedures/ OR exp. Knee/ or knee arthroplasty.mp. OR exp. Hips/ or hip arthroplasty.mp. OR shoulder surgery.mp. OR Spinal Fusion/ or spine surgery.mp. or Spine/)

## Abbreviations

CBT: Cognitive Behaviour Therapy;
CESD-10: the Centre for Epidemiologic Studies Depression scale-10.
GI: Guided Imagery;
MI: Motivational Interviewing;
PCS: Pain Catastrophizing Scale;
PE: Psycho-education;
PMP: Pain management program;
PRISMA: Preferred Reporting Items for Systematic reviews and Meta-Analyses
RCTs: Randomised Control Trials
RT: Relaxation Therapy;
Sig.: Significant;
STAI: State-Trait Anxiety Inventory;
TAU: Treatment as usual;
THA: Total Hip Arthroplasty;
TKA: Total Knee Arthroplasty;
VAS: Visual analogue scale;
WOMAC: Western Ontario and McMaster Universities Osteoarthritis Index;
